# Supplementary material for: The prognostic role of lymphocyte-to-monocyte ratio in patients with resectable pancreatic cancer: a systematic review and meta-analysis
Source: PeerJ. 2024 Jul 18;12:e17585. doi: 10.7717/peerj.17585 (PMC11260418; doi:10.7717/peerj.17585)
Supplement: Supplemental Information 2 — Pubmed, Embase, Cochrane and Web of Science [file peerj-12-17585-s002.pdf]

Pubmed-103

((((("Lymphocytes"[Mesh]) OR (((((Lymphocyte) OR (Lymphoid Cells)) OR (Cell, Lymphoid)) OR (Cells, Lymphoid)) OR (Lymphoid Cell))) AND (("Monocytes"[Mesh]) OR (Monocyte))) AND (ratio))) AND (("Pancreatic Neoplasms"[Mesh]) OR (((((((((((((((((Neoplasm, Pancreatic) OR (Pancreatic Neoplasm)) OR (Pancreas Neoplasms)) OR (Neoplasm, Pancreas)) OR (Neoplasms, Pancreas)) OR (Pancreas Neoplasm)) OR (Neoplasms, Pancreatic)) OR (Cancer of Pancreas)) OR (Pancreas Cancers)) OR (Pancreas Cancer)) OR (Cancer, Pancreas)) OR (Cancers, Pancreas)) OR (Pancreatic Cancer)) OR (Cancer, Pancreatic)) OR (Cancers, Pancreatic)) OR (Pancreatic Cancers)) OR (Cancer of the Pancreas)))

Embase-143

▼ **Search History** (1)

	# ▲	Searches	Results	Type
☐	1	((Lymphocytes or (Lymphocyte or Lymphoid Cells or Cell, Lymphoid or Cells, Lymphoid or Lymphoid Cell)) and (Monocytes or Monocyte) and ratio and (Pancreatic Neoplasms or (Neoplasm, Pancreatic or Pancreatic Neoplasm or Pancreas Neoplasms or Neoplasm, Pancreas or Neoplasms, Pancreas or Pancreas Neoplasm or Neoplasms, Pancreatic or Cancer of Pancreas or Pancreas Cancers or Pancreas Cancer or Cancer, Pancreas or Cancers, Pancreas or Pancreatic Cancer or Cancer, Pancreatic or Cancers, Pancreatic or Pancreatic Cancers or Cancer of the Pancreas))).af.	143	Advanced

Cochrane-3

▼ **Search History** (1)

	# ▲	Searches	Results	Type
☐	1	((Lymphocytes or (Lymphocyte or Lymphoid Cells or Cell, Lymphoid or Cells, Lymphoid or Lymphoid Cell)) and (Monocytes or Monocyte) and ratio and (Pancreatic Neoplasms or (Neoplasm, Pancreatic or Pancreatic Neoplasm or Pancreas Neoplasms or Neoplasm, Pancreas or Neoplasms, Pancreas or Pancreas Neoplasm or Neoplasms, Pancreatic or Cancer of Pancreas or Pancreas Cancers or Pancreas Cancer or Cancer, Pancreas or Cancers, Pancreas or Pancreatic Cancer or Cancer, Pancreatic or Cancers, Pancreatic or Pancreatic Cancers or Cancer of the Pancreas))).af.	6	Advanced

WOS-106

(((((Lymphocytes) OR (((((Lymphocyte) OR (Lymphoid Cells)) OR (Cell, Lymphoid)) OR (Cells, Lymphoid)) OR (Lymphoid Cell))) AND ((Monocytes) OR (Monocyte))) AND (ratio))) AND ((Pancreatic Neoplasms) OR (((((((((((((((((Neoplasm, Pancreatic) OR (Pancreatic Neoplasm)) OR (Pancreas Neoplasms)) OR (Neoplasm, Pancreas)) OR (Neoplasms, Pancreas)) OR (Pancreas Neoplasm)) OR (Neoplasms, Pancreatic)) OR (Cancer of Pancreas)) OR (Pancreas Cancers)) OR (Pancreas Cancer)) OR (Cancer, Pancreas)) OR (Cancers, Pancreas)) OR (Pancreatic Cancer)) OR (Cancer, Pancreatic)) OR (Cancers, Pancreatic)) OR (Pancreatic Cancers)) OR (Cancer of the Pancreas)))
