# Supplementary material for: The prognostic role of lymphocyte-to-monocyte ratio in patients with resectable pancreatic cancer: a systematic review and meta-analysis
Source: PeerJ. 2024 Jul 18;12:e17585. doi: 10.7717/peerj.17585 (PMC11260418; doi:10.7717/peerj.17585)
Supplement: Supplemental Information 3 — *indicates criterion met; - indicates significant of criterion not met. [file peerj-12-17585-s003.pdf]

Supplementary Table S3. Quality evaluation of the eligible studies with Newcastle–Ottawa scale.

	Selection				Comparability		Outcome		
Study	Representative-ness	Selection of non-exposed	Ascertainmentof exposure	Outcome not present at start	Comparability on most important factors	Comparability on other risk factors	Assessment of outcome	Long enough follow-up (median≥1 year)	Adequacy (completeness) of follow-up
Neumann , 2023 (36)	*	*	*	*	-	-	*	*	-
Kubota 2022 (35)	-	*	*	*	*	-	*	*	*
Ueberroth 2021 (34)	-	-	*	*	*	-	*	*	*
Markus 2021 (33)	-	*	*	*	-	-	*	*	*
Fang 2021 (32)	-	*	*	*	*	-	*	*	*
Zhou 2020 (31)	-	*	*	*	*	-	*	*	*
Takeuchi 2020 (30)	-	*	*	*	*	-	*	*	*
Takano 2020 (29)	-	*	*	*	*	-	*	*	*
Pointer 2020 (28)	-	*	*	*	*	-	*	*	*
Onoe 2019 (27)	-	*	*	*	-	-	*	*	*
Kawai 2019 (26)	-	*	*	*	*	-	*	-	*
Sierzega 2017 (25)	-	*	*	*	-	-	*	*	-
Li 2016 (24)	*	*	*	*	*	-	*	*	*
Stotz 2015 (23)	-	*	*	*	-	-	*	*	*

*indicates criterion met; - indicates significant of criterion not met.
