# Supplementary material for: The prognostic role of lymphocyte-to-monocyte ratio in patients with resectable pancreatic cancer: a systematic review and meta-analysis
Source: PeerJ. 2024 Jul 18;12:e17585. doi: 10.7717/peerj.17585 (PMC11260418; doi:10.7717/peerj.17585)
Supplement: Supplemental Information 4 [file peerj-12-17585-s004.doc]

Methods

Literature SearchThis systematic analysis adhered to the PRISMA (Preferred Reporting Items for Systematic Reviews and Meta-Analyses) 2020 guidelines and and relied on data registered in PROSPERO under the reference CRD42023461260. The PRISMA 2020 checklist can be found within Supplementary Table S1.As of September 2023, we conducted a systematic literature search using PubMed, Embase, Cochrane, and Web of Science to investigate the prognostic role of the monocyte-to-lymphocyte ratio in resectable pancreatic cancer, and the publications were in English. We used the following terms to search the databases: “Lymphocytes” “Lymphocyte” “Lymphoid Cells” “Cell, Lymphoid Cells” “Neoplasm, Pancreatic” “Pancreatic Neoplasm”“Pancreas Neoplasms”“Neoplasm, Pancreas” “Neoplasms, Pancreas” “Pancreas Neoplasm” “Neoplasms, Pancreatic” “Cancer of Pancreas” “Pancreas Cancers” “Pancreas Cancer”“ Cancer, Pancreas” “Cancers, Pancreas” “Pancreatic Cancer” “Cancer, Pancreatic” “Cancers, Pancreatic”“Pancreatic Cancers”“Cancer of the Pancreas”。Detailed search strategies can be found in Supplementary Table S2. The search query we use in Pubmed is like this：((((("Lymphocytes"[Mesh]) OR (((((Lymphocyte) OR (Lymphoid Cells)) OR (Cell, Lymphoid)) OR (Cells, Lymphoid)) OR (Lymphoid Cell))) AND (("Monocytes"[Mesh]) OR (Monocyte))) AND (ratio))) AND (("Pancreatic Neoplasms"[Mesh]) OR (((((((((((((((((Neoplasm, Pancreatic) OR (Pancreatic Neoplasm)) OR (Pancreas Neoplasms)) OR (Neoplasm, Pancreas)) OR (Neoplasms, Pancreas)) OR (Pancreas Neoplasm)) OR (Neoplasms, Pancreatic)) OR (Cancer of Pancreas)) OR (Pancreas Cancers)) OR (Pancreas Cancer)) OR (Cancer, Pancreas)) OR (Cancers, Pancreas)) OR (Pancreatic Cancer)) OR (Cancer, Pancreatic)) OR (Cancers, Pancreatic)) OR (Pancreatic Cancers)) OR (Cancer of the Pancreas)))The search queries for Embase, Cochrane, and WOS are as follows：(((((Lymphocytes) OR (((((Lymphocyte) OR (Lymphoid Cells)) OR (Cell, Lymphoid)) OR (Cells, Lymphoid)) OR (Lymphoid Cell))) AND ((Monocytes) OR (Monocyte))) AND (ratio))) AND ((Pancreatic Neoplasms) OR (((((((((((((((((Neoplasm, Pancreatic) OR (Pancreatic Neoplasm)) OR (Pancreas Neoplasms)) OR (Neoplasm, Pancreas)) OR (Neoplasms, Pancreas)) OR (Pancreas Neoplasm)) OR (Neoplasms, Pancreatic)) OR (Cancer of Pancreas)) OR (Pancreas Cancers)) OR (Pancreas Cancer)) OR (Cancer, Pancreas)) OR (Cancers, Pancreas)) OR (Pancreatic Cancer)) OR (Cancer, Pancreatic)) OR (Cancers, Pancreatic)) OR (Pancreatic Cancers)) OR (Cancer of the Pancreas)))Additionally, a manual review of the reference lists of all eligible studies was performed. Two investigators independently conducted searches for nested reports within primary studies (Haipeng Li and Shang Peng), each examining for potential reports within reports. If contrasting opinions arose, the two authors would engage in further discussion to reconcile differences and seek consensus. In cases where consensus was challenging to achieve, a third author (Ran An) was included to facilitate resolution.Identification of Eligible StudiesIncorporated into our analysis were studies that fulfilled the subsequent criteria: (1) employing a study design of randomized controlled trials, cohorts, or case-control studies;(2) the studies involved adult patients with pancreatic cancer; (3) the study reported LMR or Monocyte-to-Lymphocyte Ratio (MLR) data; (4) patients were eligible for surgical removal, and (5) there was sufficient data available to calculate HR.We excluded the following types of publications: reviews, letters, editorial comments, case reports, conference abstracts, pediatric articles, unpublished articles, and articles not in English. Additionally, patients with advanced pancreatic cancer were excluded, as surgical removal is not feasible for this patient population. For studies that provided MLR data, we applied a reciprocal transformation method as necessary.Data ExtractionData extraction was independently conducted by two investigators. In cases of discordance, a third investigator was consulted to arrive at a consensus and make a final determination. We extracted the following data from the studies included in our analysis: primary author, publication year, study duration, study location, sample size, participant age, and body mass index (BMI). In instances where the study reported continuous variables as a median with a range or interquartile range, we applied a validated mathematical approach to compute the mean ± standard deviation. In cases where data were absent or remained unreported within the study, we proactively reached out to the corresponding authors, seeking the provision of complete data if it was accessible.Quality AssessmentWe evaluated the quality of the incorporated studies utilizing the Newcastle–Ottawa Scale (NOS),with studies receiving seven to nine points considered as high-quality. Additionally, we appraised the level of evidence for each study following the guidelines established by the Oxford Centre for Evidence-Based Medicine Levels of Evidence Working Group. Two investigators independently appraised the quality of each eligible study and assigned an evidence level. Any disparities were resolved through discussion between the two investigators.Statistical AnalysisWe performed evidence synthesis employing Review Manager version 5.3, developed by the Cochrane Collaboration in Oxford, UK. For comparing continuous and dichotomous variables, we employed the Hazard ratio(HR) respectively. All metrics were presented with corresponding 95% confidence intervals (CIs). To assess the heterogeneity among the studies, we used the chi-squared test (Cochran’s Q) and the inconsistency index (I2) .Significant heterogeneity was indicated by a χ p-value < 0.05 or I2 > 50%. In cases where significant heterogeneity was identified, we applied a random-effects model for the estimation of the combined HR. Otherwise, a fixed-effect model was applied. Furthermore, we conducted one-way sensitivity analyses to gauge the individual study impacts on the combined results for outcomes characterized by significant heterogeneity. To assess the presence of publication bias, we generated funnel plots using Review Manager 5.3 (Cochrane Collaboration, Oxford, UK). Additionally, for outcomes that encompassed ten or more included studies, we performed Egger's regression tests using Stata version 15.0 (Stata Corp, College Station, TX, USA). A p-value < 0.05 was considered indicative of statistically significant publication bias.Contribution

In the early stages, Romualdas Riauka and colleagues reported on the prognostic significance of the Preoperative Platelet to Lymphocyte Ratio (PLR) in resectable pancreatic cancer. They concluded that PLR is a predictive factor for better Disease-Free Survival (DFS) in patients with resectable pancreatic cancer. However, the prognostic role of the Lymphocyte to Monocyte Ratio for resectable pancreatic cancer remains a subject of controversy. Our study aims to investigate whether the Lymphocyte-to-Monocyte Ratio (LMR) can serve as an indicator for predicting the prognosis of preoperative pancreatic cancer patients. We conducted a systematic literature search in PubMed, Embase, Cochrane, and Web of Science databases up to September 2023 to assess whether LMR can predict the prognosis of preoperative pancreatic cancer patients. A total of 14 eligible articles, comprising 4,019 patients, were included in our comprehensive analysis. Distinguishing our work from that of Li W et al, we reintegrated six new pancreatic cancer articles from the past six years, expanding the patient cohort by 2224 individuals. This substantial increase has significantly enhanced the accuracy of our predictive model. Additionally, we conducted subgroup analyses on different pancreatic cancer types, yielding noteworthy predictive outcomes. Diverging from the study conducted by Shuwen Lin and colleagues, our research distinguishes itself by incorporating three highly representative articles from the past four years into the analysis. Furthermore, we included discussions on regional variations, such as Asia, Europe, and the Americas, through subgroup analyses, thus enriching the meta-analysis in this field with a more comprehensive perspective.
